# Changes of serum fibroblast growth factor 23 (FGF23) and Klotho in diabetic kidney disease and their correlation with coronary artery calcification: a retrospective study

**DOI:** 10.20945/2359-4292-2026-0125

**Published:** 2026-10-11

**Authors:** Yan Hong, Jiahui Wang, Qiandi Lu, Wenjuan Sun, Jinzhu Sun

**Affiliations:** 1 Department of Nephrology, Jiangnan University Medical Center, Wuxi, Jiangsu, China; 2 Department of Nephrology, Wuxi No. 2 People's Hospital, Affiliated Wuxi Clinical College of Nantong University, Wuxi, Jiangsu, China

**Keywords:** diabetic kidney disease, fibroblast growth factor 23, Klotho, coronary artery calcification, biomarker

## Abstract

**Objective:**

This study investigated the association among fibroblast growth factor 23 (FGF23), Klotho, and coronary artery calcification (CAC) in patients with diabetic kidney disease **(DKD)**.

**Subjects and methods:**

A total of 176 DKD patients and 160 age-and diabetes-matched controls were retrospectively analyzed. We measured serum FGF23 and Klotho levels by enzyme-linked immunosorbent assay and assessed CAC by CAC scoring. Correlation and receiver operating characteristic analyses were performed to evaluate the associations of these biomarkers with CAC and their ability to discriminate CAC.

**Results:**

FGF23 levels were significantly higher and Klotho levels were lower in patients with DKD than in controls. FGF23 correlated negatively with estimated glomerular filtration rate and positively with the albumin-to-creatinine ratio and blood urea nitrogen, whereas Klotho correlated positively with estimated glomerular filtration rate and negatively with albumin-to-creatinine ratio and blood urea nitrogen. In patients with CAC, FGF23 levels were higher and Klotho levels were lower. Receiver operating characteristic analysis showed that both FGF23 and Klotho discriminated CAC, and FGF23 showed a higher area under curve than Klotho. Correlation analysis further confirmed the association of FGF23 and Klotho with CAC severity.

**Conclusion:**

FGF23 and Klotho were associated with renal impairment and CAC in patients with DKD and may serve as potential biomarkers of renal and cardiovascular risk.

## INTRODUCTION

Diabetic kidney disease (DKD) is a leading cause of end-stage renal disease, severely impairing patients' quality of life and creating a significant socioeconomic burden (^1^, ^2^). In China, the growing number of diabetic patients, driven by population aging and lifestyle changes, has significantly increased the proportion of dialysis patients with DKD and has amplified the challenges of prevention and treatment (^3^).

The pathogenesis of DKD involves factors such as glucose metabolism, oxidative stress, inflammation, and hemodynamic changes. Chronic hyperglycemia activates the polyol, protein kinase C, advanced glycation end-products and their receptor, and hexosamine pathways, increasing the production of reactive oxygen species and inducingoxidative stress (^4^). Oxidative stress in turn activates inflammatory signaling pathways, leading to immune cell infiltration, cytokine release, and renal fibrosis (^5^, ^6^). Hemodynamic factors, including glomerular hyperperfusion and hypertension, further exacerbate kidney injury (^7^). Together, these mechanisms drive pathological changes in intrinsic renal cells, including mesangial cell proliferation, basement membrane thickening, and extracellular matrix accumulation, which impair glomerular filtration. Classically, DKD presents with progressive albuminuria and declining renal function (^8^, ^9^). In recent years, this classical pattern has been challenged by the recognition of non-albuminuric DKD, a phenotype characterized by marked renal dysfunction in the absence of albuminuria (^10^). Notably, non-albuminuric DKD is increasingly common in patients with type 2 diabetes mellitus (T2DM) and has become the predominant phenotype in some cohorts (^11^).

As DKD progresses, impaired renal function directly reduces phosphate excretion and results in hyperphosphatemia. This condition stimulates parathyroid hormone (PTH) secretion and thereby induces secondary hyperparathyroidism (^12^). Additionally, the imbalance in calcium-phosphate metabolism inhibits the metabolic activation of vitamin D and reduces the synthesis of active vitamin D (^13^, ^14^). This metabolic disturbance not only exacerbates the disruption of calcium-phosphate balance but also significantly increases the risk of skeletal disorders such as osteoporosis and fracture (^15^). More importantly, disturbances in calcium-phosphate metabolism are closely associated with the cardiovascular system, particularly because they promote the onset and progression of cardiovascular disease through vascular calcification. Vascular calcification is a core pathological feature of chronic kidney disease-mineral and bone disorder; it involves dysregulation of hormones such as fibroblast growth factor 23 (FGF23) and ultimately increases cardiovascular mortality (^16^, ^17^).

FGF23 is a phosphate-regulating hormone secreted primarily by osteocytes and participates in the regulation of mineral metabolism. It maintains calcium-phosphate balance by inhibiting renal phosphate reabsorption and reducing the synthesis of active vitamin D (^18^). As renal function declines in DKD, serum FGF23 levels rise significantly. Although elevated FGF23 was initially regarded as a compensatory response that maintains phosphate balance, it is now linked to worsening renal function and to the early onset of kidney disease (^19^, ^20^). Furthermore, high FGF23 levels may accelerate disease progression by promoting inflammation and fibrosis (^21^). Elevated FGF23 independently increases the risk of cardiovascular disease and all-cause mortality, even in patients with T2DM and normal or mildly impaired renal function (^22^). In DKD, FGF23 may disrupt its interaction with Klotho, leading to “FGF23 resistance“, which further disrupts signaling pathways and accelerates the progression of kidney disease (^23^).

Klotho protein, a co-receptor for FGF23, is highly expressed in the kidney and participates in FGF23 signaling (^24^). It exists in a transmembrane form and is expressed primarily in the kidney, enhancing the affinity between FGF23 and its receptor (^25^). Transmembrane Klotho is hydrolyzed to soluble Klotho, which enters the bloodstream and exerts FGF23-independentfunctions, including anti-inflammatory and anti-fibrotic effects that protect the kidney and the cardiovascular system (^26^). In patients with DKD, serum Klotho levels are markedly reduced (^27^), and this reduction correlates negatively with the degree of renal impairment. In early chronic kidney disease (CKD), a decline in serum ot-Klotho is often accompanied by increased FGF23 levels, correlates with a decrease in estimated glomerular filtration rate (eGFR), and serves as an early biomarker of renal dysfunction (^28^). Reduced Klotho expression can disrupt FGF23 signaling. In CKD, Klotho deficiency causes FGF23 dysregulation, such as resistance or Klotho-independent effects, which worsens calcium-phosphate metabolism and renal damage (^25^). Klotho deficiency also impairs endothelial function by diminishing the protective role of soluble Klotho and by increasing oxidative stress and inflammation, thereby raising the risk of cardiovascular diseases such as heart failure and myocardial hypertrophy (^29^).

Coronary artery calcification (CAC), a manifestation of coronary atherosclerosis, is characterized by the deposition of calcium salts in the coronary artery wall. In the general population, the incidence of CAC increases with age; in patients with DKD; however, both the incidence and the severity of CAC are significantly higher than in the general population. Studies have shown that the incidence of CAC in dialysis patients can reach 80% and correlates positively with allcause mortality (^30^). CAC is driven by inflammation, endothelial injury, disorders of calcium-phosphate metabolism, and apoptosis. In patients with DKD, hyperphosphatemia and dysregulation of the FGF23-Klotho axis contribute to the progression of CAC. Overexpression of FGF23 promotes the transformation of vascular smooth muscle cells into osteoblastlike cells via the Wnt7b/β-catenin pathway and thereby exacerbates vascular calcification (^31^). Reduced Klotho levels, in turn, impair endothelial function and promote calcium-phosphate deposition in the vessel wall by increasing oxidative stress and inflammation (^32^). These mechanisms reinforce the pathological progression of CAC in DKD.

Although existing studies have highlighted the important roles of FGF23 and Klotho in both DKD and CAC, the precise relationship between these two factors and their combined contribution to the pathogenesis of DKD with concomitant CAC remains unclear. Further investigation is essential to clarify the mechanisms of DKD with CAC and could provide new insights and a theoretical foundation for early clinical diagnosis, disease monitoring, and targeted therapeutic intervention.

## SUBJECTS AND METHODS

### Study population

This retrospective study was approved by the Jiangnan University Medical Center, and informed written consent was waived. We established the diagnosis of DKD according to the Expert Consensus on Diabetic Kidney Disease (2014 edition), which defines DKD as an eGFR < 60 mL/min/1.73 m^2^ or an albumin-to-creatinine ratio (ACR) > 30 mg/g persisting for more than three months. We diagnosed T2DM according to the criteria set out in the 1999 World Health Organization guidelines. The study included 176 patients with DKD and 160 controls with T2DM matched for age, gender, and diabetes duration. Hyperlipidemia was defined according to the Chinese Guidelines for the Management of Dyslipidemia in Adults (2016 Revision) and was diagnosed when any of the following criteria were met: fastingtotal cholesterol ≥ 5.2 mmol/L, fasting triglycerides ≥ 1.7 mmol/L, fasting LDL-C ≥ 3.4 mmol/L, or current use of lipidlowering therapy, including statins or fibrates.

### Sample size estimation

Sample size was estimated based on the primary objective, namely to evaluate the ability of FGF23 and Klotho to discriminate CAC in patients with DKD. The calculation used an receiver operating characteristic (ROC) curve-based sample size formula in PASS (v. 15.0). Drawing on our preliminary data and previous reports indicating an area under curve (AUC) of approximately 0.75 for FGF23 in predicting CAC, we set the two-sided significance level at α = 0.05 and the statistical power at 1 — β = 0.80, with an expected ratio of cases (CAC group) to controls (no coronary artery calcification [NCAC] group) of approximately 1:1.75. The minimum required sample size was 58 patients in the CAC group and 102 patients in the NCAC group. We included a total of 176 patients with DKD, comprising 64 in the CAC group and 112 in the NCAC group; the sample size was therefore adequate for the planned analyses.

### Inclusion and exclusion criteria

The inclusion criteria for patients with DKD were a confirmed diagnosis of T2DM and DKD based on established clinical criteria, age ≥ 18 years, and availability of complete clinical data. For the T2DM control group without kidney disease, the inclusion criteria were a confirmed diagnosis of T2DM according to standard guidelines, age ≥ 18 years, and availability of complete clinical data. The exclusion criteria for patients with DKD and T2DM were diabetic ketoacidosis, hyperglycemic hyperosmolar syndrome, concurrent fever, infection, and acute cardiovascular or cerebrovascular events, including acute myocardial infarction, stroke, and intracerebral hemorrhage. We also excluded patients with hepatic insufficiency, rheumatic diseases, hematologic disorders, pregnancy, malignancies, fractures, or primary hyperparathyroidism, as well as those who had used glucocorticoids, immunosuppressants, or nephrotoxic drugs within the preceding three months.

### Coronary artery calcium assessments

We performed chest computed tomography (CT) in patients with DKD using a 64-slice spiral CT scanner and assessed the degree of CAC with the Agatston method. Before coronary artery calcium score (CACS) measurements, patients' heart rates were controlled, and the scan was completed in a single session whenever possible. The CACS were calculated separately for the left main coronary artery, left anterior descending artery, left circumflex artery, and right coronary artery. The total CACS was obtained by summing the scores from these four main coronary artery branches. Specifically, calcified plaques were defined as lesions with an area ≥ 1 mm^2^ and a CT density ≥ 130 HU. Each 1 mm^2^ area was considered as the scoring unit, and we multiplied the peak CT value by the calcified to calculate the calcification score. The calcification values were recorded for each layer, and the total CACS was calculated by summing the values across all layers. The CACS was calculated independently for the left main coronary artery, left anterior descending artery, left circumflex artery, and right coronary artery, and the total CACS was the sum of these individual scores. The presence of CAC was determined according to the CACS cutoff, classifying patients into a calcified group (CACS > 0) and a non-calcified group (CACS = 0).

### Serum FGF23 and Klotho measurements

We measured serum concentrations of FGF23 and Klotho using commercially available enzyme-linked immunosorbent assay (ELISA) kits (Human FGF23, EH3058; Human Klotho, EH2184; Wuhan Fein Biological Technology) and performed all measurements in strict accordance with the manufacturer's protocols.

### Statistical analysis

Correlation analyses between serum FGF23, serum Klotho, and kidney function markers were conducted, and ROC curve analysis was used to assess the ability of serum FGF23 and Klotho to discriminate CAC in patients with DKD. Before analysis, we assessed the normality of all continuous variables with the Shapiro-Wilk test. Continuous variables with an approximately normal distribution are presented as mean ± standard deviation, whereas non-normally distributed variables are expressed as median and interquartile range. Categorical variables are presented as n (%). Group comparisons were performed using the unpaired t-test with Welch's correction for normally distributed variables and the Mann-Whitney U test for non-normally distributed variables. Fisher's exact test was used for categorical variables.

## RESULTS

### Clinical data of patients with diabetes mellitus and diabetic kidney disease

We compared the clinical data of the DM and DKD groups. Age, body mass index, duration of diabetes, gender distribution, and family history of diabetes did not differ significantly between the two groups (*p* > 0.05 for all; **Table 1**). However, eGFR was markedly lower in the DKD group (53.44 ± 11.75 mL/min/1.73 m^2^) than in the DM group (124.37 ± 21.05 mL/min/1.73 m^2^; *p* < 0.001). The ACR was significantly higher in the DKD group (95.53 [60.81-135.07] mg/g) than in the DM group (12.44 [8.52-15.26] mg/g), indicating greater albuminuria in patients with DKD. Blood urea nitrogen (BUN) was likewise significantly higher in the DKD group (8.81 ± 2.52 mmol/L) than in the DM group (5.03 ± 1.18 mmol/L; *p* < 0.001). Serum phosphorus and PTH levels were also significantly higher in the DKD group than in the DM group (1.38 ± 0.20 vs. 1.06 ± 0.17 mmol/L, *p* < 0.001; 74.62 ± 24.16 vs. 48.54 ± 15.38 pg/mL, *p* < 0.001, respectively), whereas 25(OH)D levels were significantly lower (16.84 ± 5.27 vs. 22.37 ± 6.55 ng/mL; *p* < 0.001). Serum calcium did not differ significantly between the two groups (2.30 ± 0.23 vs. 2.32 ± 0.22 mmol/L; *p* = 0.425). Accordingto the KDIGO 2012 classification, the eGFR-based DKD stages were distributed as follows: G4, 3 patients (1.7%); G3b, 36 patients (20.4%); G3a, 92 patients (52.3%); and G2, 45 patients (25.6%). The albuminuria stages were distributed as follows: Al, 2 patients (1.1%); A2, 164 patients (93.2%); and A3, 10 patients (5.7%). These findings emphasized the marked biochemical changes associated with DKD compared with T2DM without kidney involvement.

**Table 1:** Clinical data between diabetes mellitus and diabetic kidney disease

**Variable**	**DM(n = 160)**	**DKD(n = 176)**	***p-*Va** lue
Age (years)	59.87 ±9.52	60.28 ±9.86	0.594
Body mass index (kg/m^2^)	25.91 ±5.02	25.53 ±4.71	0.368
Duration of DM (years)	9.45 ±2.63	9.62 ±2.79	0.627
Gender			0.584
Male	87(54.4%)	101 (57.4%)	
Female	73(45.6%)	75(42.6%)	
Family history of DM			0.762
Yes	23(14.4%)	28(15.9%)	
No	137(85.6%)	148(84.1%)	
Hypertension			0.155
Yes	42(26.3%)	59(33.5%)	
No	118(73.7%)	117(66.5%)	
Hyperlipemia			0.679
Yes	29(18.1%)	36(20.5%)	
No	131 (81.9%)	140(79.5%)	
HbA1c(%)	7.84 ±1.56	8.12 ± 1.93	0.136
eGFR(mL/min/1.73m^2^)	124.37 ±21.05	53.44 ±11.75	&lt; 0.001
ACR (mg/g)	12.44(8.52-15.26)	95.53(8.52-15.26)	&lt; 0.001
Serum calcium (mmol/L)	2.32 ±0.22	2.30 ±0.23	0.425
Serum phosphorus (mmol/L)	1.06 ±0.17	1.38 ±0.20	&lt; 0.001
Serum PTH (pg/mL)	48.54 ±15.38	74.62 ±24.16	&lt; 0.001
25(0H)D (ng/mL)	22.37 ±6.55	16.84 ±5.27	&lt; 0.001
BUN (mmol/L)	5.03 ± 1.18	8.81 ±2.52	&lt; 0.001
Serum FGF23 (pg/mL)	44.32 ±19.45	62.79 ±29.21	&lt; 0.001
Serum Klotho (pg/mL)	628.91 ±231.46	486.07 ± 228.73	&lt; 0.001

The data were presented as mean ± standard deviation or median (interquartile range) for continuous variables, and n (%) for categorical variables. The comparisons of data between the DM and DKD groups were done by the Mann-Whitney test or unpaired t-test with Welch's correction or Fisher's exact test. ACR: Albumin/creatinine ratio; BUN: blood urea nitrogen; DM: diabetes mellitus; DKD: diabetic kidney disease; eGFR: estimated glomerular filtration rate.

### Comparisons of serum FGF23 and Klotho levels between patients with diabetes mellitus and diabetic kidney disease

To further examine the differences in serum FGF23 and Klotho levels between patients with DM and those with DKD, we performed comparative analyses (**Table 1**). Serum FGF23 levels were significantly higher in the DKD group (62.79 ± 29.21 pg/mL) compared to the DM group (44.32 ± 19.45 pg/mL; *p* < 0.001; **Figure 1A**), whereas serum Klotho levels were significantly lower in patients with DKD (486.07 ± 228.73 pg/mL) than in patients with DM (628.91 ± 231.46 pg/mL; *p* < 0.001; **Figure 1B**). These results confirmed the biochemical alterations observed in DKD, namely elevated FGF23 and decreased Klotho levels, suggesting that these markers may be associated with renal dysfunction in DKD.

**Figure 1:**
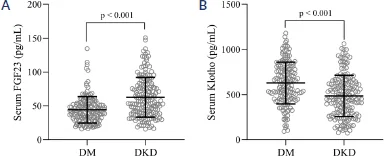
Comparisons of serum FGF23 (A) and Klotho (B) between patients with diabetes mellitus (DM, n = 160) and diabetic kidney disease (DKD, n = 176). The data were presented as mean ± standard deviation. *p*-Values were calculated from an unpaired t-test with Welch's correction.

### Correlation of serum FGF23 and Klotho with eGFR, albumin/creatinine ratio, and blood urea nitrogen

To investigate the association between serum FGF23 and Klotho levels and renal function in DKD, we conducted correlation analyses in 176 patients, relating serum FGF23 and Klotho concentrations to the renal function markers eGFR, ACR, and BUN. Serum FGF23 correlated significantly and negatively with eGFR (r = -0.39, *p* < 0.001), indicating that higher FGF23 levels were associated with worsening kidney function **(Figure 2A**). In contrast, FGF23 correlated significantly and positively with both ACR (r = 0.44, p < 0.001; **Figure 2B**) and BUN (r = 0.35, *p* < 0.001; **Figure 2C**), suggesting that elevated FGF23 was linked to increased albuminuria and higher BUN levels, both markers of kidney damage. Conversely, serum Klotho correlated positively with eGFR (r = 0.33, *p* < 0.001; **Figure 2D**), indicating that higher Klotho levels were associated with better kidney function, and correlated significantly and negatively with ACR (r = -0.40, *p* < 0.001) and BUN (r = -0.37, *p* < 0.001), suggesting that elevated Klotho levels were linked to reduced albuminuria and lower BUN levels (Figure 2E-F). These findings support the association of FGF23 and Klotho with kidney function and renal injury in DKD.

**Figure 2:**
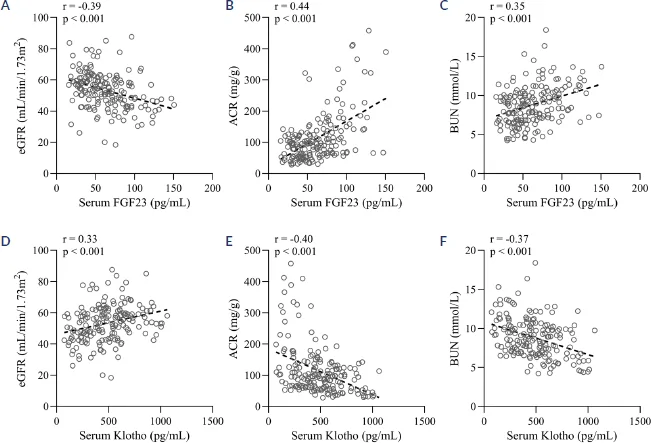
Correlation analysis of serum FGF23 with estimated glomerular filtration rate (eGFR) (A), albumin-to-creatinine ratio (ACR) (B), blood urea nitrogen (BUN) (C); serum Klotho with eGFR (D), ACR (E), BUN (F) in patients with diabetic kidney disease (n = 176).

### Clinical data of patients with diabetic kidney disease with and without coronary artery calcification

We next compared the clinical characteristics of patients with DKD with and without CAC. The two groups differed significantly in several respects (**Table 2**). Patients in the CAC group were older (62.14 ± 10.05 vs. 58.83 ± 9.72 years; *p* < 0.001) and had a longer duration of diabetes (10.76 ± 2.98 vs. 8.38 ± 2.76 years; *p* < 0.001). Hyperlipidemia was more prevalent in the CAC group (31.2% vs. 14.3%; p = 0.011), and HbA1c levels were higher (8.47 ± 1.92% vs. 7.65 ± 1.88%; *p* < 0.001). The CAC group also had significantly higher serum phosphorus and PTH levels than the NCAC group (1.43 ± 0.21 vs. 1.34 ± 0.19 mmol/L, *p* = 0.008; 81.29 ± 25.43 vs. 70.82 ± 22.56 pg/mL, *p* = 0.007, respectively), together with significantly lower 25(OH)D levels (15.64 ±4.82 vs. 17.55 ± 5.48 ng/ ml_; *p* = 0.021). body mass index, eGFR, ACR, and BUN did not differ significantly between the groups, nor did systolic or diastolic blood pressure (140.51 ± 16.24 vs. 138.26 ± 15.67 mmHg, *p* = 0.395; 83.16 ± 9.72 vs. 82.59 ± 9.34 mmHg, *p* = 0.508, respectively). The use of lipid-lowering therapy and of RAS blockers was also comparable between the groups (62.5% vs. 58.9%, *p* = 0.749; 70.3% vs. 60.7%, p = 0.253, respectively).

**Table 2:** Clinical data between coronary artery calcification or not in patients with diabetic kidney disease

**Variable**	**NCAC(n = 112)**	**CAC(n = 64)**	***p-*Value 1**
Age (years)	58.83 ±9.72	62.14 +10.05	&lt; 0.001
Body mass index (kg/m^2^)	25.69 ±4.90	25.37 ±4.85	0.448
Duration of DM (years)	8.38 ±2.76	10.76 ±2.98	&lt; 0.001
Gender			0.206
Male	60(53.6%)	41(64.1%)	
Female	52(46.4%)	23(35.9%)	
Family history of DM			0.831
Yes	17(15.2%)	11(17.2%)	
No	95(84.8%)	53(82.8%)	
Hypertension			0.139
Yes	33(29.5%)	26(40.6%)	
No	79(70.5%)	38(59.4%)	
Hyperlipemia			0.011
Yes	16(14.3%)	20(31.2%)	
No	96(85.7%)	44(68.8%)	
Lipid-lowering medications			0.749
Yes	66(58.9%)	40(62.5%)	
No	46(41.1%)	24(37.5%)	
RAS blockers			0.253
Yes	68(60.7%)	45(70.3%)	
No	44(39.3%)	19(29.7%)	
SBP(mmHg)	140.51 ±16.24	138.26 ±15.67	0.395
DBP(mmHg)	83.16 ±9.72	82.59 ±9.34	0.508
HbA1c(%)	7.65 ±1.88	8.47 ±1.92	&lt; 0.001
eGFR(mL/min/1.73m^2^)	51.19 ± 11.63	55.04 ±11.81	0.267
ACR (mg/g)	93.67(45.72-136.28)	98.07(48.36-159.04)	0.315
BUN(mmol/L)	8.57 ±2.49	9.02 ±2.55	0.196
Serum calcium (mmol/L)	2.29 ±0.24	2.31 ±0.23	0.346
Serum phosphorus (mmol/L)	1.34 + 0.19	1.43 ±0.21	0.008
Serum PTH (pg/mL)	70.82 ±22.56	81.29 ±25.43	0.007
25(0H)D (ng/mL)	17.55 ±5.48	15.64 ±4.82	0.021
Serum FGF23 (pg/mL)	52.42 ±22.12	80.96 ±31.33	&lt; 0.001
Serum Klotho (pg/mL)	545.87 ± 223.24	381.41 ±200.13	&lt; 0.001

The data were presented as mean ± standard deviation or median (interquartile range) for continuous variables, and n (%) for categorical variables. The comparisons of data between the DM and DKD groups were done by Mann-Whitney test or unpaired t-test with Welch's correction or Fisher's exact test. ACR: Albumin/creatinine ratio; BUN: blood urea nitrogen; DBP: diastolic blood pressure; DM: diabetes mellitus; DKD: diabetic kidney disease; eGFR: estimated glomerular filtration rate; SBP: systolic blood pressure.

### Comparisons of serum FGF23 and Klotho levels between patients with diabetic kidney disease with and without coronary artery calcification

To examine the relationship between serum FGF23 and Klotho levels and the presence of CAC in patients with DKD, we compared these biomarkers between 64 patients with CAC and 112 patients without CAC (NCAC). Serum FGF23 levels were significantly higher in the CAC group (80.96 ± 31.33 pg/mL) than in the NCAC group (52.42 ± 22.12 pg/mL; *p* < 0.001; **Figure 3A).** Conversely, serum Klotho levels were significantly lower in the CAC group (381.41 ± 200.13 pg/mL) than in the NCAC group (545.87 ± 223.24 pg/mL; *p* < 0.001; **Figure 3B).** These findings suggested that elevated FGF23 and reduced Klotho levels were associated with the presence of CAC in patients with DKD.

**Figure 3:**
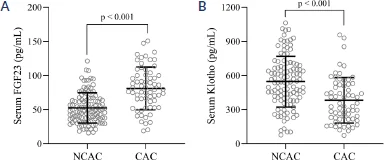
Comparisons of serum FGF23 (**A**) and Klotho (**B**) between coronary artery calcification (CAC; n = 64) or not (NCAC; n = 112) in patients with diabetic kidney disease. The data were presented as mean ± standard deviation. *p*-Values were calculated from an unpaired t-test with Welch's correction.

### Multivariate logistic regression analysis for coronary artery calcification in patients with diabetic kidney disease

To determine whether FGF23 and Klotho were independently associated with CAC in patients with DKD, we performed multivariate logistic regression analysis with adjustment for age, duration of diabetes, hyperlipidemia, HbA1c, eGFR, ACR, serum calcium, phosphorus, and PTH (**Table 3**). Both serum FGF23 and Klotho remained independently associated with the presence of CAC: higher FGF23 levels were associated with a greater likelihood of CAC (OR 1.029, 95% Cl 1.011-1.047; *p* = 0.002), whereas higher Klotho levels were associated with a lower likelihood of CAC (OR 0.996, 95% CI 0.993-0.999; p = 0.015). Age, duration of diabetes, hyperlipidemia, HbAlc, and PTH were also independently associated with CAC.

**Table 3:** Multivariate logistic regression analysis for coronary artery calcification in patients with diabetic kidney disease

**Variable**	**OR (95% Cl)**	*p*-Value
Age (per 1-year increase)	1.052(1.013-1.092)	0.008
Duration of DM (per 1-year increase)	1.125(1.047-1.209)	0.001
Hyperlipemia (yes = 1)	1.876(1.023-3.442)	0.042
HbAlc (per 1% increase)	1.213(1.035-1.421)	0.017
eGFR (per 1 ml_/min/1.73m^2^ increase)	0.987(0.962-1.013)	0.315
ACR (per 10mg/g increase)	1.004(0.998-1.010)	0.186
Serum calcium (per 0.1 mmol/L increase)	1.085(0.912-1.291)	0.342
Serum phosphorus (per 0.1 mmol/L increase)	1.215(0.987-1.496)	0.066
PTH (per 1 pg/mL increase)	1.018(1.003-1.033)	0.018
Serum FGF23 (per 1 pg/mL increase)	1.029(1.011-1.047)	0.002
Serum Klotho (per 1 pg/mL increase)	0.996 (0.993-0.999)	0.015

Multivariate logistic regression analysis was performed to identify independent predictors of CAC in DKD patients. Variables with *p&lt;* 0.1 in univariate analysis were initially considered for inclusion, along with clinically established confounders (eGFR, ACR, serum calcium, serum phosphorus, and PTH) that are associated with CAC in DKD populations. Continuous variables were entered as per-unit increments as specified, and categorical variables were coded as binary variables. Multicollinearity was evaluated using variance inflation factor (VIF), and all VIF values were &lt; 5, confirming no significant multicollinearity among independent variables. A full-variable enter method was used to construct the final model to minimize selection bias. ACR: Albumin/creatinine ratio; CI: confidence interval; DM: diabetes mellitus; eGFR: estimated glomerular filtration rate; FGF23: fibroblast growth factor 23; HbA1 c: glycated hemoglobin A1 c; OR: odds ratio; PTH: parathyroid hormone.

### ROC Analysis of the predictive value of serum FGF23 and Klotho for coronary artery calcification

To assess the predictive value of serum FGF23 and Klotho levels for CAC in patients with DKD, we performed ROC curve analysis (**Figure 4**). Serum FGF23 had a higher AUC of 0.77 (95% CI 0.69-0.84; *p* < 0.001), with a sensitivity of 71.88% and a specificity of 75.00%, corresponding to a Youden index of 0.47 (**Table 4**). Serum Klotho demonstrated an AUC of 0.72 (95% CI 0.64-0.79; *p* < 0.001), with a sensitivity of 68.75% and a specificity of 66.96%, corresponding to a Youden index of 0.36 (Table 4). These results indicated that both FGF23 and Klotho had moderate discriminatory ability for identifying CAC in patients with DKD, with FGF23 showing a numerically higher AUC than Klotho.

**Table 4:** Predictive values of serum FGF23 and Klotho for coronary artery calcification in patients with diabetic kidney disease

**Variable**	**Cut off (pg/mL)**	**AUC (95% CI)**	***p*-Value**	**Sensitivity**	**Specificity**	**Yl**
Serum FGF23	64.94	0.77 (0.69-0.84)	&lt; 0.001	71.88%	75.00%	0.47
Serum Klotho	449.03	0.72 (0.64-0.79)	&lt; 0.001	68.75%	66.96%	0.36

AUC: area under curve; CI: confidence interval; YI: Youden index.

**Figure 4:**
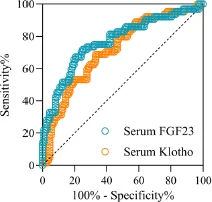
ROC analysis for predictive values of serum FGF23, Klotho for coronary artery calcification in patients with diabetic kidney disease.

### Correlation of coronary artery calcification scores with serum FGF23 and Klotho in patients with diabetic kidney disease and coronary artery calcification

To investigate the association between serum FGF23 and Klotho levels and the severity of CAC in patients with DKD, we analyzed the correlations between CACS and serum FGF23 and Klotho concentrations. Serum FGF23 correlated significantly and positively with CACS (r = 0.58, p < 0.001), indicating that higher FGF23 levels were associated with greater CAC severity (Figure 5A). Conversely, serum Klotho correlated significantly and negatively with CACS (r = -0.55, p < 0.001), suggesting that greater CAC severity was associated with lower Klotho levels (Figure 5B). Collectively, these findings support the association of FGF23 and Klotho with CAC burden in patients with DKD.

**Figure 5:**
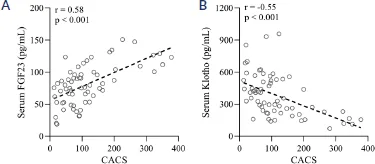
Spearman correlation analysis of coronary artery calcification score (CACS) with serum FGF23 (A) and Klotho (B) in diabetic kidney disease patients with coronary artery calcification (n = 64).

## DISCUSSION

This study compared clinical data between patients with DM and patients with DKD, revealing significant changes in FGF23 and Klotho levels in patients with DKD, and explored the relationship of these markers with CAC. FGF23 levels were significantly higher in patients with DKD than in patients with DM, while Klotho levels were significantly lower in patients with DKD, suggesting that these biochemical markers may be associated with renal dysfunction in DKD.

With respect to renal impairment, FGF23 levels correlated negatively with eGFR and positively with both ACR and BUN, suggesting that elevated FGF23 is closely associated with declining kidney function and worsening kidney injury. Conversely, Klotho levels correlated positively with eGFR and negatively with both ACR and BUN, indicating that reduced Klotho may be associated with deteriorating kidney function and aggravated renal injury. These findings further support an association of FGF23 and Klotho with renal dysfunction in patients with DKD.

In the analysis of CAC, serum FGF23 levels were significantly higher in patients with CAC than in those without CAC, whereas serum Klotho levels were significantly lower. These findings suggest that elevated FGF23 and reduced Klotho may be closely associated with the presence of CAC in patients with DKD. Considered together with the differences in serum phosphorus, PTH, and 25(OH)D between the CAC and NCAC groups, these data further support a close association between disordered mineral metabolism and vascular calcification in DKD.

In the analysis of CAC severity, serum FGF23 levels correlated positively with CAC scores, whereas serum Klotho levels correlated negatively. These findings are biologically plausible, because disturbances in the FGF23-Klotho axis have been closely associated with vascular calcification in CKD (^26^). In CKD, Klotho deficiency promotes vascular calcification by increasing phosphate uptake and mineralization in vascular smooth muscle cells (^33^). Vascular Klotho deficiency may also accelerate calcification through Runx2- and myocardin-serum response factor-dependent pathways (^34^). Emerging evidence further suggests that high FGF23 may act directly on the vasculature by driving a phenotypic switch of vascular smooth muscle cells from a contractile to a synthetic state, thereby contributing to arterial remodeling and stiffness, although the direct vascular actions of FGF23 remain under debate (^34^, ^35^).

Our findings are consistent with the existing literature, demonstrating that elevated FGF23 levels and decreased Klotho levels in patients with DKD are associated with deteriorating renal function and cardiovascular risk. Previous studies have reported that, in CKD, serum FGF23 levels rise while soluble Klotho levels decrease, and that these changes serve as early biomarkers of renal dysfunction, correlating with declining eGFR and proteinuria (e.g., ACR) (^28^, ^36^). For instance, a decrease in Klotho followed by an increase in FGF23 is regarded as an early biomarker of renal dysfunction and can predict cardiovascular risk (^37^). Regarding cardiovascular implications, both FGF23 and Klotho are closely associated with CAC (^38^). Our findings suggest that FGF23 and Klotho reflect related but distinct dimensions of cardiovascular injury in DKD. Although both belong to the same biological axis, they appear to participate in partly different pathophysiological processes in CKD and cardiovascular disease (^26^). FGF23 may be more closely linked to disordered mineral metabolism and cardiovascular risk, whereas Klotho may better reflect the loss of endogenous vascular protection and anti-calcific capacity (^26^). Combined assessment of the two markers may therefore provide complementary information for cardiovascular risk stratification in DKD.

The novelty of this study lies in the integrated assessment of FGF23 and Klotho in relation to both renal dysfunction and CAC in DKD. Beyond their associations with kidney injury, both biomarkers were independently associated with the presence of CAC, suggesting that they reflect complementary aspects of cardiovascular injury in DKD. From a clinical perspective, FGF23 and Klotho are better regarded as adjunctive rather than stand-alone biomarkers. Previous studies have linked elevated FGF23 to cardiovascular events and mortality in patients with T2DM (^19^), whereas reduced soluble Klotho has been associated with poorer renal and extra-renal outcomes in DKD (^27^); these findings support the potential clinical relevance of our results. Nevertheless, the discriminatory ability was only moderate. Prospective multicenter studies are needed to determine whether adding FGF23 and Klotho to conventional clinical variables improves risk stratification and clinical decision-making.

This study has several limitations. First, the cross-sectional design prevents establishing causal relationships between elevated FGF23, reduced Klotho, and the progression of DKD or CAC; longitudinal studies are needed to explore these connections over time. Second, despite the adequate sample size, generalizability may be limited, especially across different ethnic groups or DKD stages. Third, because of the retrospective design, we could not systematically collect data on diabetic retinopathy, peripheral neuropathy, detailed lipid profiles including LDL-C and HDL-C, family history of premature cardiovascular disease, or smoking status for all participants, which may have resulted in residual confounding. Fourth, we assessed CAC using clinical measures, whereas advanced imaging techniques could provide more accurate results. Lastly, further research is required to fully elucidate the molecular mechanisms underlying the observed associations.

In conclusion, this study showed that elevated FGF23 and reduced Klotho levels were associated with renal impairment and CAC in patients with DKD. These findings support the potential relevance of both markers as indicators of renal and cardiovascular risk. Nevertheless, further investigations are warranted to validate their clinical applicability and to elucidate the underlying pathophysiological mechanisms.

## Data Availability

data are available upon reasonable request.
